# Characteristics of Pediatric Allergic Rhinitis With Different Disease Severity

**DOI:** 10.1155/mi/5553039

**Published:** 2025-01-16

**Authors:** Yinhui Zeng, Ting Lin, Wanhua Xie, Shengli Gao, Qingxiang Zeng, Xi Luo, Wenlong Liu

**Affiliations:** ^1^Department of Otolaryngology, Guangzhou Women and Children's Medical Center, Guangzhou Medical University, Guangzhou 510623, China; ^2^Department of Orthodontics, Affiliated Stomatology Hospital of Guangzhou Medical University, Guangdong Engineering Research Center of Oral Restoration and Reconstruction, Guangzhou Key Laboratory of Basic and Applied Research of Oral Regenerative Medicine, Guangzhou 510182, China; ^3^Outpatient Department, Guangzhou Women and Children's Medical Center, Guangzhou Medical University, Guangzhou 510623, China

**Keywords:** allergic rhinitis, children, severity

## Abstract

**Background:** Although numerous studies have focused on diagnostic biomarkers to help identify allergic rhinitis (AR), data on the characteristics of pediatric AR with different severity is limited. We aimed to compare the characteristics of pediatric AR with different severity.

**Methods:** A total of 1054 children with AR were enrolled and classified into mild intermittent AR, mild persistent AR, moderate-to-severe intermittent AR, and moderate-to-severe persistent AR. All children were surveyed using a questionnaire that included detailed demographic information. Blood cell analysis was performed using an automatic hematology analyzer.

**Results:** No significant differences were observed in feeding patterns, dietary habits, outdoor activity time, total IgE, eosinophil count, and eosinophil percentage among the different AR subgroups. However, a higher prevalence of a family history of AR was noted in the moderate-to-severe persistent group. Symptoms were more likely to exacerbate when using air conditioning in children with moderate-to-severe persistent AR. Multivariate regression analysis also showed that symptom exacerbation when using air conditioning was associated with disease severity.

**Conclusions:** This study suggests that exacerbation of symptoms during air conditioning use can be considered a predictive factor for the severity of pediatric AR. Doctors and parents should pay special attention to these children to prevent more severe symptoms and improve the quality of life of these patients as early as possible.

## 1. Introduction

Allergic rhinitis (AR) is an IgE-mediated nasal mucosal immune disease caused by common allergens, characterized by increased mucus secretion and airway hyperreactivity [1]. In China, the incidence of AR has increased with the acceleration of industrialization and urbanization [2]. Population-based cross-sectional surveys conducted in various regions of China have revealed that the prevalence of AR among adults varies between 9.6% and 23.9%, with an average prevalence rate of 9.8% observed among children [3, 4]. Although AR is not life-threatening, its severity significantly impacts the quality of life of children [5].

In the Allergic Rhinitis and its Impact on Asthma (ARIA) Guidelines, experts have suggested a straightforward qualitative assessment approach to categorize AR severity based on quality-of-life factors such as sleep, learning, and physical activities. This method divides children with AR into two groups: mild (where none of these factors are affected) and moderate-to-severe (where one or more of these factors are impacted) [6].

Although numerous studies have focused on diagnostic biomarkers to help identify AR, data on the characteristics of pediatric AR with different severity is limited [7–9]. We aimed to compare the characteristics of pediatric AR with different severity.

## 2. Materials and Methods

### 2.1. Study Subjects

A total of 1054 children with comprehensive AR data, who visited our hospital between August 2015 and August 2023, were consecutively enrolled in the study. Our study has received approval from the Ethics Committee of our hospital, and all children's guardians were required to assist in completing the informed consent form. The inclusion criteria encompassed the following: the presence of typical nasal symptoms in accordance with the ARIA Guidelines, a positive serum-specific IgE test or skin prick test for at least one type of aeroallergen (including house dust mites [HDM], cockroaches, weed pollen, fungi, cat dander, and dog dander), and an age range of 3–18 years. Children with incomplete clinical data, tumors, mental disorders, or other allergic (asthma, atopic dermatitis) and systemic diseases were excluded from the study.

AR was classified into mild intermittent AR, mild persistent AR, moderate-to-severe intermittent AR, and moderate-to-severe persistent AR based on ARIA Guidelines. Information on gender, age, height, weight, family history, dietary habits, living environment, and other relevant factors were collected.

### 2.2. Total Nasal Symptom and Medication Scores

The total nasal symptom score encompassed four symptoms: nasal itchiness, nasal congestion, sneezing, and rhinorrhea. Each symptom was rated on a scale from 0 to 3 based on subjective severity. A total score of >0 and ≤4 was defined as mild AR, while a score of >4 and ≤12 was defined as moderate-to-severe AR [10].

The medication scores were assigned as follows: 0 points for no medication use, 1 point for oral and/or topical antihistamines, 2 points for nasal corticosteroid spray, and 3 points for oral corticosteroids. The sum of all medication records constituted the total medication score.

### 2.3. Laboratory Test

Serum total IgE and allergen-specific IgE were measured using the ELISA method with the Phadia UniCAP 100 analyzer (Sweden). Blood cells were analyzed by an automatic hematology analyzer.

### 2.4. Statistical Analysis

Statistical analysis was done by SPSS version 23 (SPSS, Chicago, USA). Normally distributed continuous variables were described as mean ± standard deviation and analyzed by the *t*-test. Categorical variables were presented as rates and percentages and analyzed by the chi-square test. Logistic regression analysis was performed to explore the factors affecting the severity of AR. A *p*-value less than 0.05 indicated statistical significance.

## 3. Results

### 3.1. Demographic Characteristics

Our study comprised 1054 pediatric patients with AR, of which 718 (68.1%) were male and 336 (31.9%) were female. Based on the ARIA classification, 111 patients (10.5%) were diagnosed with mild intermittent rhinitis, 91 patients (8.6%) with moderate-to-severe intermittent rhinitis, 339 patients (32.2%) with mild persistent rhinitis, and 513 patients (48.7%) with moderate-to-severe persistent rhinitis (Table 1). There were no statistically significant differences in age, gender composition ratio, and BMI among different groups.

### 3.2. Comparison of Symptoms and Medication Scores Between Different Among Various Subgroups

The moderate-to-severe group exhibited significantly higher nasal symptom scores, ocular symptom scores, total symptom scores, and symptom medication scores compared to the mild group. Although the medication scores were also higher in the moderate-to-severe group, this difference did not reach statistical significance (Table 1).

### 3.3. Comparison of Dietary Factors Among Various Subgroups

No significant differences were observed in feeding methods, dietary habits (frequency of seafood, poultry, and pork consumption), or outdoor activity time among the various subgroups of AR (Table 2).

### 3.4. Comparison of Genetic and Environmental Factors Among Various Subgroups

A higher proportion of children with a family history of AR was observed in the moderate-to-severe persistent group. Symptoms were more likely to exacerbate when using air conditioning in children with moderate-to-severe persistent AR. However, no statistically significant differences were found in living environment, region, pet, and plush toy ownership, passive smoking history, air conditioning usage frequency, bedding cleaning frequency, bedroom occupancy time, and carpet ownership among the various subgroups of AR (Table 3). Multivariate regression analysis revealed that only symptom exacerbation when using air conditioning was significantly associated with disease severity (Table 4).

### 3.5. Comparison of Hematological-Related Indicators Among Various Subgroups

No significant differences were observed in total IgE, eosinophil count, and eosinophil percentage among various subgroups of AR (Table 5).

## 4. Discussion

In recent years, the incidence of pediatric AR has been on the rise annually, fueled by rapid economic development and increasing environmental changes. An epidemiological study conducted in 2005, utilizing cluster stratified sampling methods, surveyed 23,791 children aged 6–13 years across eight provincial capital cities in four regions. The results revealed that the average prevalence of AR among these children was 9.8% [4]. Additionally, a cross-sectional survey conducted between October 2008 and May 2009 found that the self-reported prevalence of AR was 14.46%, 20.42%, and 7.83% in Beijing, Chongqing, and Guangzhou, respectively [11]. AR significantly impacts children's health and quality of life and poses a substantial burden on society, making it a global health concern.

The clinical manifestations of AR vary significantly. Mild cases may occur sporadically, with minimal impact on daily life and learning, whereas severe cases can impair breathing and sleep, resulting in lethargy and a substantial decline in quality of life. Consequently, identifying the factors that contribute to the progression of childhood AR from mild to severe holds significant clinical and research value.

Consistent with previous studies, our data revealed that the incidence of AR among boys was significantly higher than among girls, but there was no difference among subgroups, indicating that boys and girls have an equivalent risk of developing severe AR [4]. According to the ARIA classification, only 10.5% of our study population had mild intermittent rhinitis, one-third had mild persistent rhinitis, and over half suffered moderate-to-severe AR, highlighting the significant impact of AR on the majority of children. Although almost all of our subjects were sensitized to mites, ~20% still experienced seasonal symptoms, potentially due to variations in indoor allergen concentrations resulting from seasonal changes in air humidity in Guangzhou.

All nasal symptoms intensified with increasing severity, with severe nasal congestion (a score of 2 or above) affecting nearly 70% of children with moderate-to-severe AR. This suggests that nasal congestion is a prominent manifestation of severe AR in pediatric patients. Interestingly, most children with AR across various severity levels in our study displayed mild ocular pruritus symptoms. This finding may be attributed to our study population consisting of children from southern China, who are predominantly sensitized to mites. Conversely, children sensitized to pollen are more prone to developing ocular pruritus symptoms. Consistent with our results, previous studies have also demonstrated that nasal symptoms are more pronounced in children sensitive to HDM, whereas pollen allergy tends to cause ocular and systemic symptoms [12]. Regarding medication use, although the medication scores were higher in the moderate-to-severe group compared to the mild group, the difference did not reach statistical significance.

A systematic review conducted by Obbagy et al. [13] revealed insufficient and inconsistent evidence to support a correlation between feeding patterns, dietary habits, and the development of AR. Similarly, our study found no association between infant feeding patterns, current dietary habits, and the severity of childhood AR. Moreover, we did not observe significant differences in outdoor activity time among various subgroups.

The occurrence of AR is strongly linked to genetics, with studies indicating a higher risk for children whose parents have AR [14, 15]. This is especially notable in moderate-to-severe AR cases. Our findings also showed a higher proportion of children with a family history of AR in the moderate-to-severe persistent group compared to mild intermittent AR, although there were no significant differences among other groups. However, multivariate regression analysis revealed that family history was not a statistically significant factor.

We found that living environment (flat/apartment) and region (urban/rural) have no significant impact on AR severity, which may be attributed to the fact that ~90% of our subjects come from urban areas and apartments. Environmental factors, such as pets, plush toys, passive smoking, air-conditioner use, bedding cleaning frequency, bedroom duration, and carpet use, did not significantly differ among AR subgroups. Furthermore, total IgE, eosinophil count, and eosinophil percentage were similar across subgroups, indicating that AR severity in children may be more related to local immune changes than systemic changes.

We found that the exacerbation of symptoms during air-conditioner use was the only factor associated with AR severity. Previous studies have identified air-conditioner use in summer is a risk factor for the occurrence and progression of AR [16]. However, some studies suggest air-conditioners protect against wheezing by reducing outdoor allergen exposure [17, 18]. Given mites are a major allergen in southern China; air-conditioner use may expose children with AR to higher mite levels. Therefore, doctors and parents should closely monitor children with AR whose symptoms worsen when using an air-conditioner, as this may indicate a higher initial severity.

This study also has several limitations. First, its single-center design may introduce bias. Second, the focus on mite-allergic children limits the generalizability of findings to regions where pollen or other allergens dominate. Third, some relevant factors were not examined and require further research.

In conclusion, our findings suggest that symptom exacerbation during air-conditioner use can predict AR severity in children. Therefore, doctors and parents should closely monitor such children to prevent symptom progression and improve their quality of life.

## Figures and Tables

**Table 1:** Demographic characteristics and symptom drug scores of allergic rhinitis in children with different severity.

Characteristics	AR (*n* = 1054)	Mild intermittent (*n* = 111)	Mild persistent (*n* = 339)	Moderate-to-severe intermittent (*n* = 91)	Moderate-to-severe persistent (*n* = 513)	*p*
Age (years)	7.29 ± 2.544	7.18 ± 2.405	7.19 ± 2.477	7.66 ± 2.596	7.31 ± 2.606	0.109
Sex	—	—	—	—	—	0.722
Male	718 (68.1)	71 (64.0)	230 (67.8)	61 (67.0)	356 (69.4)	—
Female	336 (31.9)	40 (36.0)	109 (32.2)	30 (33.0)	157 (30.6)	—
Weight	26.95 ± 10.606	26.96 ± 11.971	27.18 ± 10.680	28.05 ± 10.722	26.60 ± 10.232	0.208
Height	126.85 ± 16.360	126.28 ± 16.769	127.10 ± 16.185	127.17 ± 16.160	126.75 ± 16.465	0.917
BMI	16.29 ± 3.775	16.26 ± 3.623	16.42 ± 4.149	17.05 ± 4.625	16.08 ± 3.347	0.718
Allergen						
Derf	896 (85.0)	84 (75.7)	316 (93.2)	82 (90.1)	414 (80.7)	0.31
Derp	785 (74.5)	65 (58.6)	308 (90.9)	65 (71.4)	347 (67.6)	0.23
Cockroach	69 (6.5)	17 (15.3)	12 (3.5)	14 (15.4)	26 (5.1)	0.06
Cat hair	52 (4.9)	12 (10.8)	15 (4.4)	11 (12.1)	14 (2.7)	0.35
Dog hair	83 (7.9)	21 (18.9)	27 (8.0)	19 (20.9)	16 (3.1)	0.12
Fungus	62 (5.9)	15 (13.5)	18 (5.3)	22 (24.2)	7 (1.4)	0.36
Weed pollen	34 (3.2)	11 (9.9)	9 (2.7)	6 (6.6)	8 (1.6)	0.51
Seasonal symptoms	—	—	—	—	—	0.192
None	418 (39.7)	48 (43.2)	147 (43.4)	36 (39.6)	187 (36.5)	—
Yes	636 (60.3)	63 (56.8)	192 (56.6)	55 (60.4)	326 (63.5)	—
Nasal itching	—	—	—	—	—	**<0.001**
0: Slight	470 (44.6)	64 (57.7)	174 (51.3)	39 (42.9)	193 (37.6)	—
1: Moderately	376 (35.7)	34 (30.6)	114 (33.6)	35 (38.5)	193 (37.6)	—
2: Severe	200 (19.0)	13 (11.7)	50 (14.7)	16 (17.6)	121 (23.6)	—
3: Unbearable	8 (0.8)	0 (0.0)	1 (0.3)	1 (1.1)	6 (1.2)	—
Sneeze	—	—	—	—	—	**<0.001**
0: None	53 (5.0)	9 (8.1)	14 (4.1)	4 (4.4)	26 (5.1)	—
1: 1–5 times	500 (47.4)	60 (54.1)	194 (57.2)	37 (40.7)	209 (40.7)	—
2: 6–10 times	307 (29.1)	32 (28.8)	85 (25.1)	32 (35.2)	158 (30.8)	—
3: ＞11 times	194 (18.4)	10 (9.0)	46 (13.6)	18 (19.8)	120 (23.4)	—
Runny nose	—	—	—	—	—	**<0.001**
0: None	187 (17.7)	32 (28.8)	65 (19.2)	15 (16.5)	75 (14.6)	—
1: 1−5 times	448 (42.5)	62 (55.9)	161 (47.5)	38 (41.8)	187 (36.5)	—
2: 6−10 times	199 (18.9)	12 (10.8)	56 (16.5)	19 (20.9)	112 (21.8)	—
3: ＞11 times	220 (20.9)	5 (4.5)	57 (16.8)	19 (20.9)	139 (27.1)	—
Nasal obstruction	—	—	—	—	—	**<0.001**
0: None	69 (6.5)	16 (14.4)	36 (10.6)	3 (3.3)	14 (2.7)	—
1: Mild	405 (38.4)	60 (54.1)	165 (48.7)	27 (29.7)	153 (29.8)	—
2: Moderately	503 (47.7)	29 (26.1)	123 (36.3)	51 (56.0)	300 (58.5)	—
3: Severe	77 (7.3)	6 (5.4)	15 (4.4)	10 (11.0)	46 (9.0)	—
TNSS	5.35 ± 2.083	4.06 ± 1.805	4.78 ± 1.984	5.68 ± 1.903	5.96 ± 2.005	**<0.001**
Eye itching	—	—	—	—	—	**0.016**
0: Slight	757 (71.8)	91 (82.0)	260 (76.7)	63 (69.2)	343 (66.9)	—
1: Moderately	189 (17.9)	14 (12.6)	56 (16.5)	17 (18.7)	102 (19.9)	—
2: Severe	102 (9.7)	6 (5.4)	21 (6.2)	11 (12.1)	64 (1.5)	—
3: Unbearable	6 (0.6)	0 (0.0)	2 (0.6)	0 (0.0)	4 (0.4)	—
Total symptom score	5.74 ± 2.327	4.30 ± 1.933	5.08 ± 2.188	6.11 ± 2.052	6.43 ± 2.278	**<0.001**
Medication score	1.02 ± 0.905	0.87 ± 0.906	0.99 ± 0.902	1.12 ± 0.905	1.06 ± 0.905	0.147
Symptom-medication score	6.76 ± 2.505	5.17 ± 2.157	6.07 ± 2.358	7.23 ± 2.251	7.49 ± 2.432	**<0.001**

*Note*: Bold for significant differences.

Abbreviation: AR, allergic rhinitis.

**Table 2:** Differences in feeding patterns, diet, or exercise in children with allergic rhinitis of different severity.

Items	AR (*n* = 1054)	Mild intermittent (*n* = 111)	Mild persistent (*n* = 339)	Moderate-to-severe intermittent (*n* = 91)	Moderate-to-severe persistent (*n* = 513)	*p*
Feeding method	—	—	—	—	—	0.155
Powdered milk	383 (36.3)	39 (35.1)	128 (37.8)	33 (36.3)	183 (35.7)	—
Breast milk	623 (59.1)	62 (55.9)	198 (58.4)	51 (56.0)	312 (60.8)	—
Combinations	48 (4.6)	10 (9.0)	13 (3.8)	7 (7.7)	18 (3.5)	—
Seafood	—	—	—	—	—	0.468
None	781 (74.1)	82 (73.9)	241 (71.1)	69 (75.8)	389 (75.8)	—
Yes	273 (25.9)	29 (26.1)	98 (28.9)	22 (24.2)	124 (24.2)	—
Poultry	—	—	—	—	—	0.924
None	435 (41.3)	46 (41.4)	135 (39.8)	39 (42.9)	215 (41.9)	—
Yes	619 (58.7)	65 (58.6)	204 (60.2)	52 (57.1)	298 (58.1)	—
Pork	—	—	—	—	—	—
None	139 (13.2)	10 (9.0)	38 (11.2)	11 (12.1)	80 (15.6)	0.136
Yes	915 (86.8)	101 (91.0)	301 (88.8)	80 (87.9)	433 (84.4)	—
Outdoor activity time (h/day)	—	—	—	—	—	0.095
≤1 h	471 (44.7)	44 (39.6)	142 (41.9)	44 (48.4)	241 (47.0)	—
2–3 h	404 (38.3)	47 (42.3)	125 (36.9)	30 (33.0)	202 (39.4)	—
>3 h	179 (17.0)	20 (18.0)	72 (21.2)	17 (18.7)	70 (13.6)	—

Abbreviation: AR, allergic rhinitis.

**Table 3:** Differences in genetic and environmental factors of allergic rhinitis in children with different severity.

Factors	AR (*n* = 1054)	Mild intermittent (*n* = 111)	Mild persistent (*n* = 339)	Moderate-to-severe intermittent (*n* = 91)	Moderate-to-severe persistent (*n* = 513)	*p*
Parents' allergy history	—	—	—	—	—	**0.049**
None	444 (42.1)	60 (54.1)	143 (42.2)	38 (41.8)	203 (39.6)	—
Yes	610 (57.9)	51 (45.9)	196 (57.8)	53 (58.2)	310 (60.4)	—
Type of residence	—	—	—	—	—	0.057
Single-story house	44 (4.2)	1 (0.9)	15 (4.4)	8 (8.8)	20 (3.9)	—
Multi-story building	1010 (95.8)	110 (99.1)	324 (95.6)	83 (91.2)	493 (96.1)	—
Residential area	—	—	—	—	—	0.354
Rural	107 (10.2)	15 (13.5)	38 (11.2)	10 (11.0)	44 (8.6)	—
Urban	947 (89.8)	96 (86.5)	301 (88.8)	81 (89.0)	469 (91.4)	—
Pet ownership	—	—	—	—	—	0.774
None	982 (93.2)	106 (95.5)	315 (92.9)	84 (92.3)	477 (93.0)	—
Yes	72 (6.8)	5 (4.5)	24 (7.1)	7 (7.7)	36 (7.0)	—
Plush toy ownership	—	—	—	—	—	0.822
None	293 (27.8)	32 (28.8)	92 (27.1)	22 (24.2)	147 (28.7)	—
Yes	761 (72.2)	79 (71.2)	247 (72.9)	69 (75.8)	366 (71.3)	—
Parents' smoking habits	—	—	—	—	—	0.653
None	681 (64.6)	70 (63.1)	228 (67.3)	59 (64.8)	324 (63.2)	—
Yes	373 (35.4)	41 (36.9)	111 (32.7)	32 (35.2)	189 (36.8)	—
Air conditioner usage frequency	—	—	—	—	—	0.067
<3 months/year	82 (7.8)	2 (1.8)	29 (8.6)	10 (11.0)	41 (8.0)	—
≥3 months/year	972 (92.2)	109 (98.2)	310 (91.4)	81 (89.0)	472 (92.0)	—
Symptoms worsen with air conditioner usage	—	—	—	—	—	**<0.001**
None	318 (30.2)	49 (44.1)	123 (36.3)	27 (29.7)	119 (23.2)	—
Yes	736 (69.8)	62 (55.9)	216 (63.7)	64 (70.3)	394 (76.8)	—
Bedding washing frequency (times/month)	—	—	—	—	—	0.948
≤1 time/month	362 (34.3)	39 (35.1)	118 (34.8)	33 (36.3)	172 (33.5)	—
>1 time/month	692 (65.7)	72 (64.9)	221 (65.2)	58 (63.7)	341 (66.5)	—
Time spent in bedroom (h)	—	—	—	—	—	0.592
<12 h/day	846 (80.3)	93 (83.8)	271 (79.9)	76 (83.5)	406 (79.1)	—
≥12 h/day	208 (19.7)	18 (16.2)	68 (20.1)	15 (16.5)	107 (20.9)	—
Carpet	—	—	—	—	—	0.052
None	955 (90.6)	108 (97.3)	307 (90.6)	79 (86.8)	461 (89.9)	—
Yes	99 (9.4)	3 (2.7)	32 (9.4)	12 (13.2)	52 (10.1)	—

*Note*: Bold for significant differences.

Abbreviation: AR, allergic rhinitis.

**Table 4:** Multivariate regression analysis.

Items	OR	Lower limit of 95% CI	Upper limit of 95% CI	*p*
Parents' allergy history	0.806	−0.448	0.015	0.067
Symptoms worsen with air conditioner usage	0.524	−0.893	−0.399	**<0.001**

*Note*: Bold for significant differences.

**Table 5:** Comparison of hematological-related indexes among different subgroups of AR.

Indexes	AR (*n* = 1054)	Mild intermittent (*n* = 111)	Mild persistent (*n* = 339)	Moderate-to-severe intermittent (*n* = 91)	Moderate-to-severe persistent (*n* = 513)	*p*
Total IgE	235 (18–873)	315 (25–694)	278 (81–561)	261 (18–873)	342 (17–476)	0.12
EOS counts	121 (11–1370)	89 (60–950)	133 (5–1370)	157 (22–795)	53 (45–813)	0.071
Proportion of EOS	2.3 (0.4–4.7)	1.6 (0.2–2.8)	1.3 (0.5–4.1)	0.9 (0.4–3.8)	1.9 (0.8–3.6)	0.36

Abbreviation: AR, allergic rhinitis.

## Data Availability

The data that support the findings of this study are available on request from the corresponding author. The data are not publicly available due to privacy or ethical restrictions.
